# TIGAR knockdown radiosensitizes TrxR1-overexpressing glioma *in vitro* and *in vivo* via inhibiting Trx1 nuclear transport

**DOI:** 10.1038/srep42928

**Published:** 2017-03-24

**Authors:** Yushuo Zhang, Fei Chen, Guomei Tai, Jiaojiao Wang, Jun Shang, Bing Zhang, Ping Wang, Baoxing Huang, Jie Du, Jiahua Yu, Haowen Zhang, Fenju Liu

**Affiliations:** 1 School of Radiation Medicine and Protection and Jiangsu Provincial Key Laboratory of Radiation Medicine and Protection, Medical College of Soochow University, Suzhou 215123, China; 2 Collaborative Innovation Center of Radiation Medicine of Jiangsu Higher Education Institutions and School for Radiological and Interdisciplinary Sciences (RAD-X), Soochow University, Suzhou 215123, China; 3 Department of Radiation Oncology, Nantong Tumor Hospital, Affiliated Tumor Hospital of Nantong University, Nantong 226321, China; 4 Institute of Radiation Medicine, Fudan University, Shanghai 200032, China

## Abstract

The up-regulation of thioredoxin reductase-1 (TrxR1) is detected in more than half of gliomas, which is significantly associated with increased malignancy grade and recurrence rate. The biological functions of NADPH-dependent TrxR1 are mainly associated with reduced thioredoxin-1 (Trx1) which plays critical roles in cellular redox signaling and tumour radio-resistance. Our previous work has proved that *TP53* induced glycolysis and apoptosis regulator (TIGAR) knockdown could notably radiosensitize glioma cells. However, whether TrxR1-overexpressing glioma cells could be re-radiosensitized by TIGAR silence is still far from clear. In the present study, TrxR1 was stably over-expressed in U-87MG and T98G glioma cells. Both *in vitro* and *in vivo* data demonstrated that the radiosensitivity of glioma cells was considerably diminished by TrxR1 overexpression. TIGAR abrogation was able to radiosensitize TrxR1-overexpressing gliomas by inhibiting IR-induced Trx1 nuclear transport. Post-radiotherapy, TIGAR low-expression predicted significant longer survival time for animals suffering from TrxR1-overexpessing xenografts, which suggested that TIGAR abrogation might be a promising strategy for radiosensitizing TrxR1-overexpressing glial tumours.

Gliomas which consist primarily of astrocytomas and oligodendrogliomas are one of the most lethal cancers in central nervous system due to their invasive and heterogeneous nature, in addition to the resistance to multimodal treatments^1^. An analysis of 433 astrocytomas has indicated that thioredoxin reductase 1 (TrxR1) becomes up-regulated in more than 66% cases, which is significantly associated with higher proliferation activity and worse prognosis^2^. Another study demonstrates that the immunoreactivity for TrxR1 is observed in more than 50% of recurrent oligodendroglial tumours, which is 1.5 times of that in primary ones, indicating that TrxR1 plays a facilitative role in the malignant progression of oligodendrogliomas^3^.

Mammalian TrxR1 is the pivotal enzyme of the thioredoxin (Trx) system which mainly comprises thioredoxin-1 (Trx1), TrxR1 and NADPH^4^. The dithiol moieties of Trx1 are reduced by receiving electrons from NADPH in the presence of TrxR1^5^. Reduced Trx1 in turn reduces downstream proteins and plays vital roles in regulating cellular redox state, inhibiting apoptosis and increasing the resistance of cancer cells to cytotoxic drugs^6^,^7^. Nevertheless, when Trx1 becomes over-oxidized, the cellular conditions may become more sensitive to oxidative stress and more inclined to apoptosis^8^. Since more than one half of glioma patients are suffered from TrxR1 overexpression^2^,^3^, there becomes pressing need for effective treatments targeting TrxR1-overexpressing gliomas.

*TP*53 induced glycolysis and apoptosis regulator (TIGAR) has recently been proved as a target gene of p53 and a regulator of cellular redox balance, whose structure and functions show similarity to fructose 2,6-bisphosphatase (FBPase-2)^9^,^10^. By inhibiting glycolysis and activating pentose phosphate pathway (PPP), TIGAR could increase the cellular NADPH level and protect cells from oxidative or metabolic stress-induced cell death^11^,^12^,^13^. Our previous study has demonstrated that TIGAR knockdown significantly radiosensitizes A172 and T98G glioma cells by depleting NADPH and postponing the reducing process of Trx1^14^. However, whether TrxR1-overexpressing glioma cells could be re-sensitized by TIGAR knockdown is still poorly understood.

This study demonstrates that U-87MG and T98G glioma cells become more invasive and more radioresistant by TrxR1 overexpression. Interestingly, TrxR1-overexpressing glioma cells are apparently re-radiosensitized by TIGAR silence. Using TrxR1-overexpressing xenograft models, it is revealed that TIGAR interfering is able to diminish the radioresistance, thus prolonging the survival time of nude mice bearing TrxR1-overexpressing gliomas notably. The mechanism is mainly correlated with NADPH depletion and the blockage of IR-induced Trx1 nuclear translocation caused by TIGAR low-expression.

## Results

### TrxR1 overexpression diminishes the radiosensitivity of glioma cells

**Figure 1:**
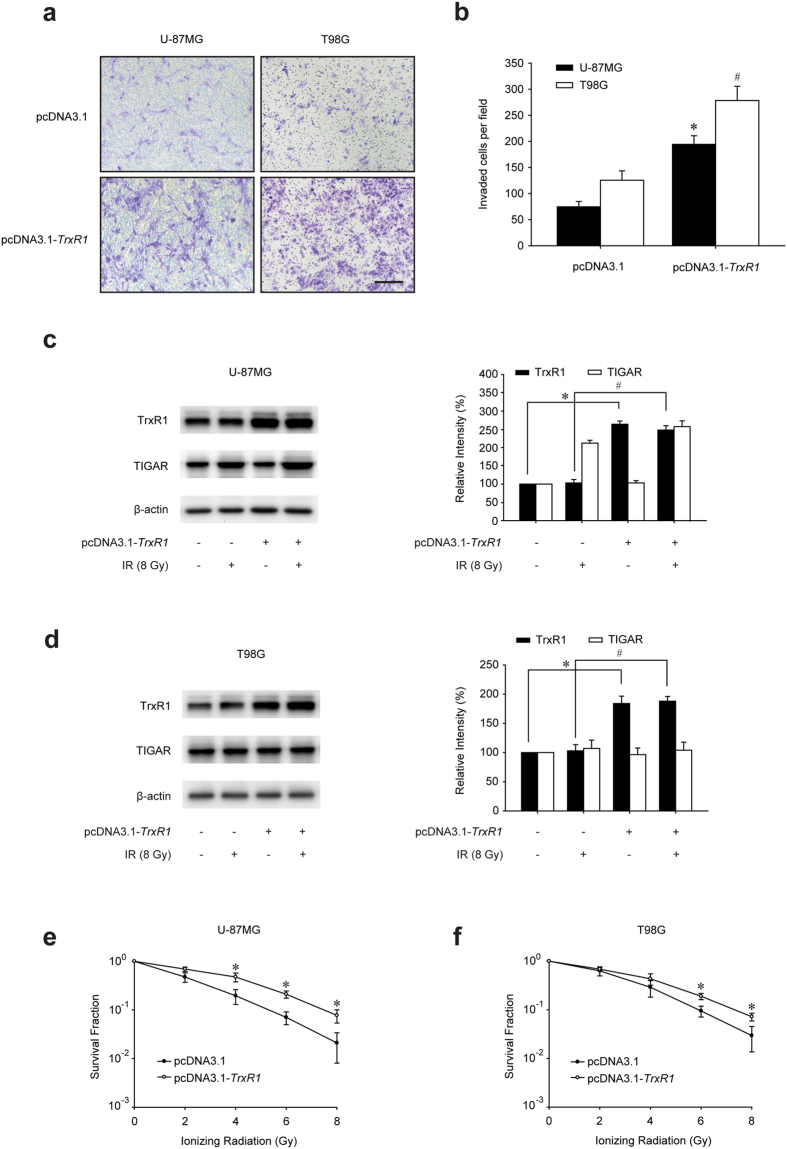
TrxR1 overexpression enhances the radioresistance of glioma cells. (**a**) Representative images of invaded U-87MG and T98G cells stably expressing pcDNA3.1 or pcDNA3.1-*TrxR1*. Cells migrated to the bottom chamber were stained with crystal violet and observed under a microscope (scale bar = 200 μm). (**b**) Invasion was quantified by determining the total number of cells that had migrated through the membrane. Accompanying statistical plots were presented to indicate statistical significance between pcDNA3.1 transfected cells and pcDNA3.1-*TrxR1* transfected cells. *p < 0.05, U-87MG cells, ^#^*p* < 0.05, T98G cells. (**c**,**d**) U-87MG and T98G cells were treated with 8 Gy irradiation 2 h before extraction, and the expression levels of TrxR1 and TIGAR was examined by Western blot (*p < 0.05, ^#^*p* < 0.05). (**e**,**f**) Clonogenic capacity of U-87MG and T98G cells with or without TrxR1 overexpression was investigated after a range of radiation doses. *p < 0.05, pcDNA3.1 vs. pcDNA3.1-*TrxR1*.

Trx1 overexpression was proved to increase tumour proliferation and invasion^15^,^16^,^17^,^18^. Nevertheless, the roles of TrxR1 in it were not fully understood. In this study, TrxR1 was demonstrated to accelerate the proliferation of U-87MG and T98G glioma cells (see Supplementary Fig. S1). Meanwhile the invasive ability of both glioma cells was considerably stimulated by TrxR1 overexpression (Fig. 1a). The numbers of invaded U-87MG and T98G glioma cells were increased to approximately 3 and 2 times respectively when TrxR1 was overexpressed (Fig. 1b). Furthermore, the radiosensitivity regulated by TrxR1 was also examined. Western blot assay revealed an increased level of TrxR1 by pcDNA3.1-*TrxR1* transfection in both U-87MG and T98G glioma cells (Fig. 1c and d). TIGAR expression was only activated in U-87MG cells 2 h post-IR but not in p53-mutant (M237I) T98G cells. Importantly, the radiosensitivity of both p53-wild type and p53-mutant glioma cells was notably diminished by TrxR1 overexpression (Fig. 1e and f).

### TIGAR abrogation radiosensitizes TrxR1-overexpressing glioma cells

**Figure 2:**
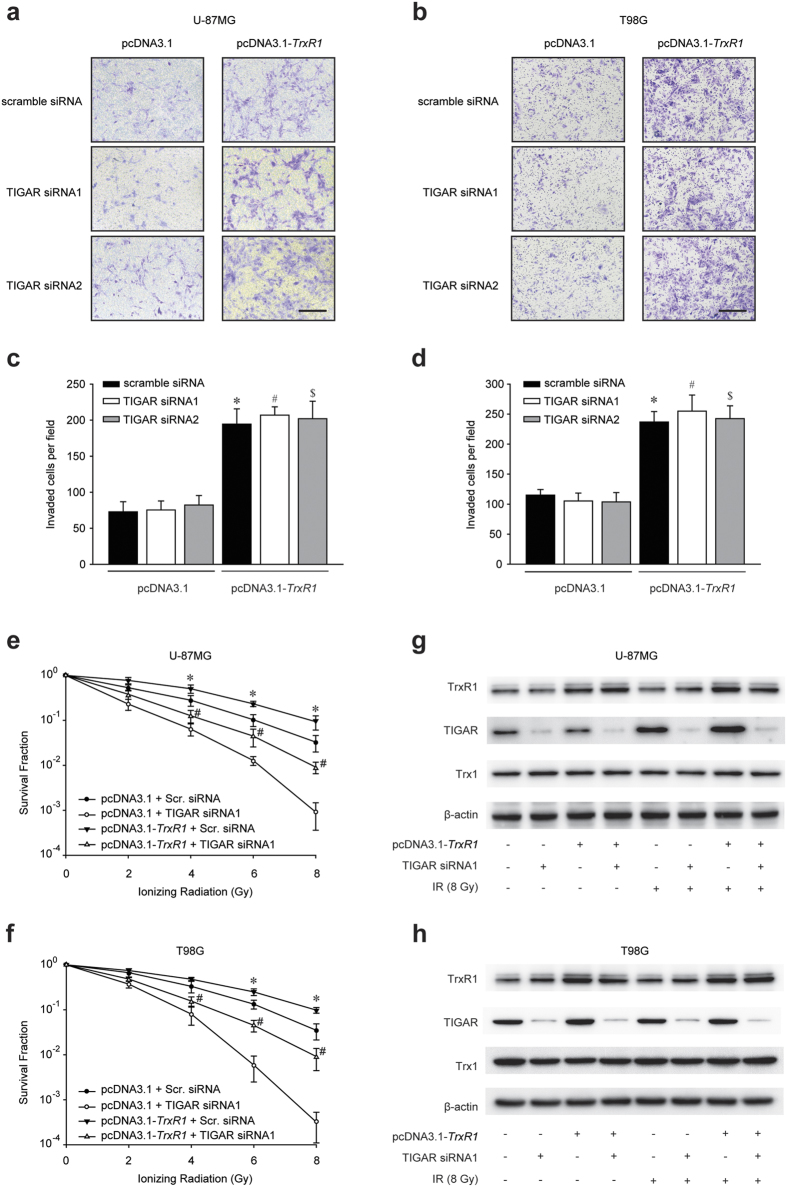
TIGAR interfering results in radiosensitization of glioma cells with TrxR1 overexpression. (**a**,**b**) Representative images of invaded U-87MG and T98G cells (scale bar = 200 μm). Cells overexpressing TrxR1 were treated with TIGAR siRNAs 48 h before inoculating into the matrigel. Accompanying statistical plots were presented to indicate statistical significance. *p < 0.05, pcDNA3.1+ scramble siRNA vs. pcDNA3.1-*TrxR1*+ scramble siRNA, ^#^*p* < 0.05, pcDNA3.1+ TIGAR siRNA1 vs. pcDNA3.1-*TrxR1*+ TIGAR siRNA1, ^$^*p* < 0.05, pcDNA3.1+ TIGAR siRNA2 vs. pcDNA3.1-*TrxR1*+ TIGAR siRNA2. (**c**,**d**) Clonogenic capacity of TrxR1-overexpressing U-87MG and T98G cells. Scramble (Scr.) or TIGAR siRNA transfection was performed 48 h before irradiation. *p < 0.05, pcDNA3.1-*TrxR1*+ Scr. siRNA vs. pcDNA3.1-*TrxR1*+ TIGAR siRNA, ^#^*p* < 0.05, pcDNA3.1+ Scr. siRNA vs. pcDNA3.1-*TrxR1*+ TIGAR siRNA. (**e**,**f**) Western blot analysis of protein expression levels in U-87MG and T98G cells. Cells were transfected with TIGAR siRNA 48 h before IR and underwent 8-Gy irradiation 2 h before being extracted.

Our previous study demonstrated that TIGAR interfering was able to radiosensitize glioma cells^14^. In this study, the effects of TIGAR knockdown on TrxR1-overexpressing glioma cells were investigated. TIGAR silence exhibited no effect on cellular proliferation (see Supplementary Fig. S2) and invasion (Fig. 2a and b) in glioma cells regardless of TrxR1 expression. Nevertheless, the radioresistance of TrxR1-overexpressing U-87MG and T98G glioma cells was dramatically diminished by TIGAR interfering (Fig. 2e,f, Supplementary Fig. S3a and b). Most noticeably, the clonogenic capacity of cells co-treated with TrxR1 overexpression and TIGAR abrogation was substantially lower than that of control cells, indicating a significant radiosensitization of TrxR1-overexpressing glioma cells. However, the total expression level of Trx1 was not able to be affected by either TIGAR interfering or irraidation (Fig. 2g,h, Supplementary Fig. S3c and S3d).

### TIGAR silence inhibits IR-induced Trx1 nuclear translocation in glioma cells with TrxR1 overexpression

**Figure 3:**
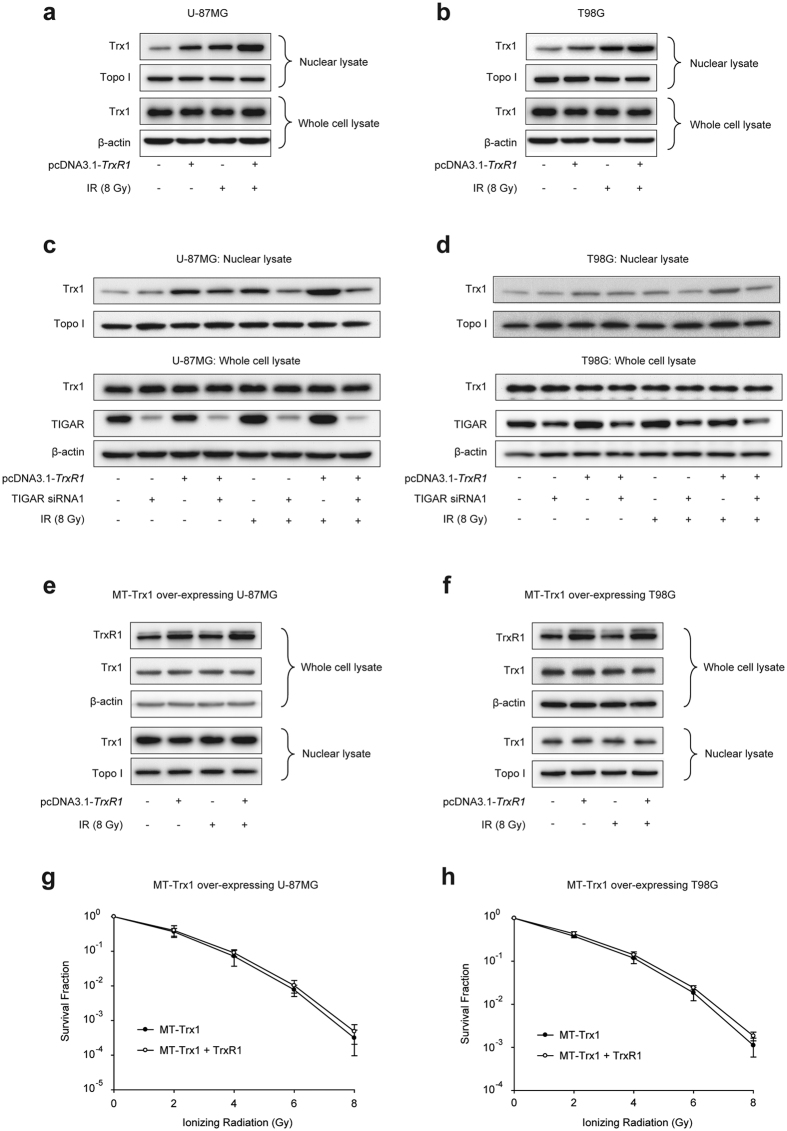
TIGAR knockdown observably abolishes IR-induced Trx1 nuclear translocation in TrxR1-overexpressing glioma cells. (**a**,**b**) Western blot analysis for nuclear lysates and whole cell lyastes of U-87MG and T98G cells. Cells were extracted 2 h post sham irradiation or 8-Gy IR. (**c**,**d**) U-87MG and T98G cells were transfected with TIGAR siRNA1 48 h before IR and underwent 8-Gy irradiation 2 h before being extracted. The expression levels of nuclear Trx1 and total Trx1 were examined by Western blot. (**e**,**f**) Western blot analysis for the cell lysates of Trx1-mutant U-87MG and T98G cells. Cells were transfected with pcDNA3.1 or pcDNA3.1-*TrxR1* 48 h before IR and underwent 8-Gy irradiation 2 h before being extracted. (**g**,**h**) Clonogenic capacity of mutant (MT)-Trx1-overexpressing U-87MG and T98G cells. Cells were transfected with pcDNA3.1 or pcDNA3.1-*TrxR1* 48 h before irradiation.

Trx1 has been reported to redistribute at nucleus 2 h post irradiation in HeLa cells, where reduced Trx1 activates the activator protein-1 (AP-1) and redox-sensitive signals^19^. Our data revealed that both pcDNA3.1-*TrxR1* transfection and irradiation could increase Trx1 nuclear translocation in glioma cells. IR-induced Trx1 nuclear expression level in TrxR1-overexpressing cells was even higher than that in parental cells (Fig. 3a and b). TIGAR abrogation seemed to have little effect on the basal level of nuclear Trx1 regardless of TrxR1 expression, as shown in Fig. 3c and d. However, in IR-exposed glioma cells, Trx1 nuclear translocation was notably diminished by TIGAR knockdown not only in pcDNA3.1 transfected cells but in TrxR1-overexpressing ones. To further demonstrate whether TrxR1 overexpression-induced radioresistance of glioma cells was dependent on the content of nuclear Trx1, glioma cells with stable overexpression of mutant type (MT)-Trx1 (K81E/K82E) was used. In MT-Trx1-overexpressing glioma cells, the nuclear translocation sequence of Trx1 was mutated and Trx1 could not transport into nucleus no matter TrxR1 was overexpressed or not (Fig. 3e and f). The clonogenic survival assay revealed that, when Trx1 lost its capacity of nuclear transport, TrxR1 overexpression could not enhance the radioresistance of glioma cells any more (Fig. 3g and h). However, in wild type (WT)-Trx1-overexpressing glioma cells, both IR-induced Trx1 nuclear translocation and the radioresistance were further increased by TrxR1 transfection (see Supplementary Fig. S4).

### TIGAR knockdown disturbs the pro-oxidant–antioxidant balance in TrxR1-overexpressing glioma cells post IR

**Figure 4:**
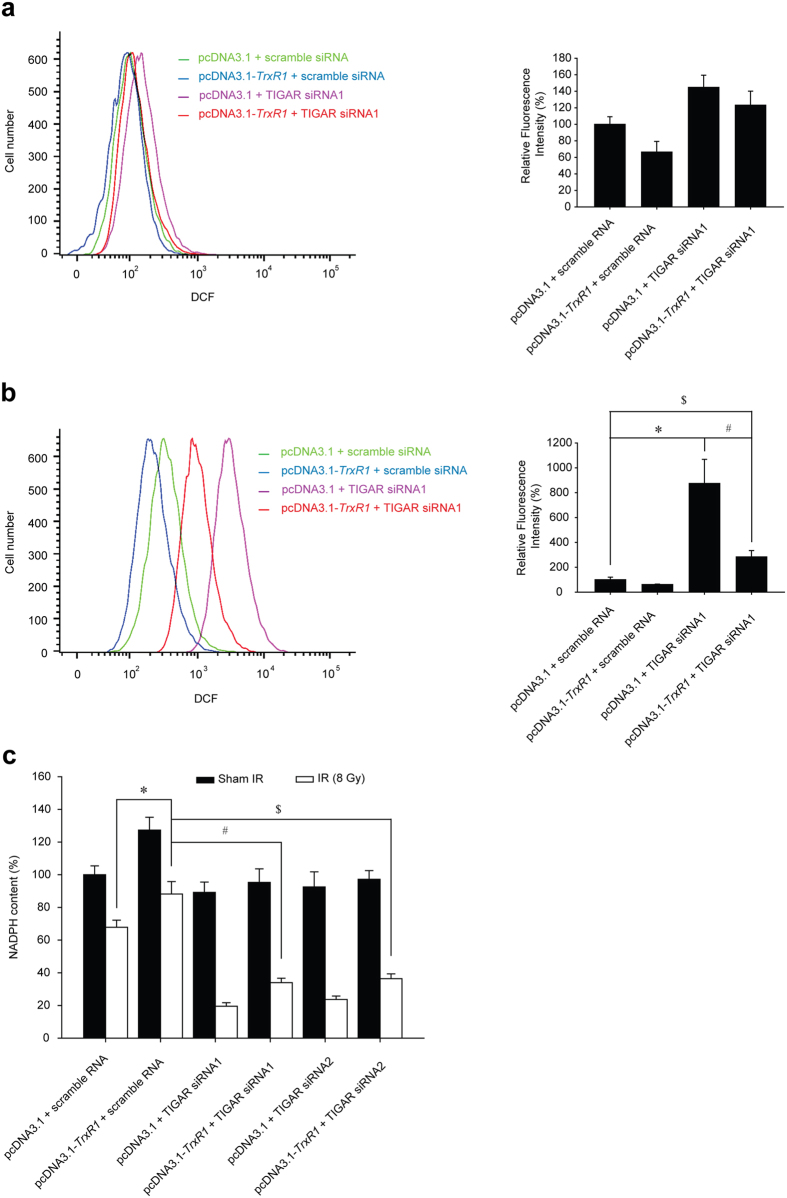
TIGAR silencing aggravates IR-induced oxidative stress in TrxR1-overexprssing glioma cells. (**a**) U-87M cells overexprssing TrxR1 were transfected with scramble siRNA or TIGAR siRNA1. Flow cytometric assessment of ROS production was performed 48 h post-transfection. (**b**) U-87MG cells were transfected with TIGAR siRNA1 48 h before IR and underwent 8-Gy irradiation 1 h before flow cytometric assessment. *p < 0.05, pcDNA3.1+ scramble siRNA vs. pcDNA3.1+ TIGAR siRNA1, ^#^*p* < 0.05, pcDNA3.1+ TIGAR siRNA1 vs. pcDNA3.1-*TrxR1*+ TIGAR siRNA1, ^$^*p* < 0.05, pcDNA3.1+ scramble siRNA vs. pcDNA3.1-*TrxR1*+ TIGAR siRNA1. (**c**) TrxR1-overexpressing U-87MG cells were transfected with TIGAR siRNA1 or TIGAR siRNA2 48 h before IR. Cellular NADPH production was measured 1 h post-IR. *p < 0.05, pcDNA3.1+ scramble siRNA vs. pcDNA3.1-*TrxR1*+ scramble siRNA, ^#^*p* < 0.05, pcDNA3.1-*TrxR1*+ sramble siRNA vs. pcDNA3.1-*TrxR1*+ TIGAR siRNA1, ^$^*p* < 0.05, pcDNA3.1-*TrxR1*+ scramble siRNA vs. pcDNA3.1-*TrxR1*+ TIGAR siRNA2.

The activity of Trx1 is correlated with the level of ROS and cellular NADPH^20^,^21^. In the present study, the ROS levels in cells treated with TrxR1 overexpression or/and TIGAR abrogation was measured by flow cytometric analysis. TIGAR inhibition alone induced a mild increase in ROS level, while TrxR1 overexpression slightly scavenged ROS no matter whether TIGAR was silenced or not (Fig. 4a and Supplementary Fig. S5a). When U-87MG glioma cells were treated with 8 Gy ionizing radiation, the ROS level was dramatically increased to 8 times by TIGAR knockdown. Although TrxR1 overexpression notably scavenged the ROS in TIGAR abrogated cells, the ROS level remained 2 times more than that in cells treated with irradiation only (Fig. 4b and Supplementary Fig. S5b). Meanwhile, TrxR1 overexpression increased the cellular NADPH content by approximately 30% in irradiated glioma cells (Fig. 4c), indicating a greater demand for NADPH as electron donors. Importantly, TIGAR knockdown induced dramatic NADPH depletion in irradiated glioma cells no matter whether TrxR1 was overexpressed or not.

### TIGAR silencing postpones the process of DNA damage repair (DDR) in TrxR1-overexpressing glioma cells

**Figure 5:**
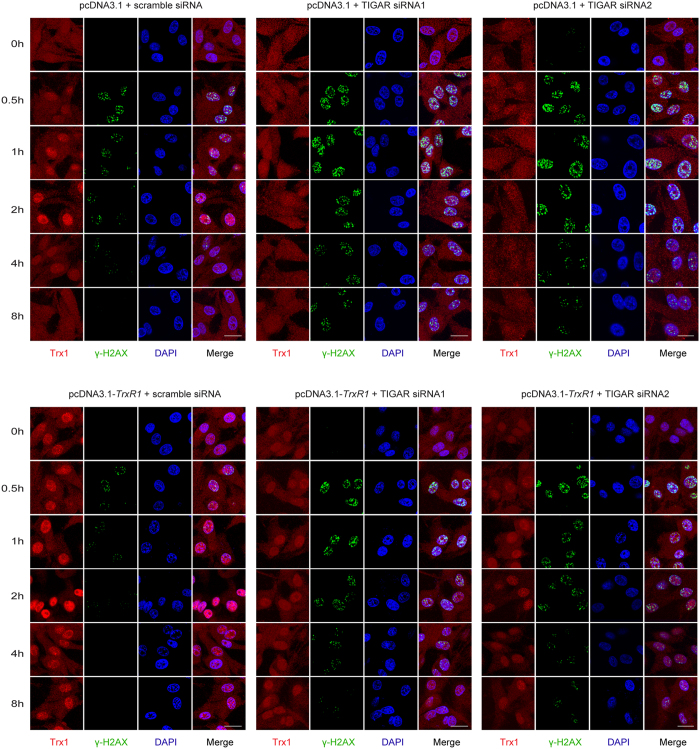
The DNA damage repair process accelerated by TrxR1 overexpression was postponed by TIGAR interfering in irradiated glioma cells. U-87MG cells stably overexpressing pcDNA3.1 or pcDNA3.1-*TrxR1* were transfected with scramble siRNA or TIGAR siRNAs 48 h before IR. Indirect immunofluorescence staining of glioma cells with anti-Trx1 and anti-γ-H2AX antibody was performed. Probe for Trx1 locus is labeled in red and γ-H2AX is labeled in green with 600x magnification (scale bar = 20 μm).

In order to further demonstrate the mechanism of TIGAR knockdown-induced radiosensitization in TrxR1-overexpressing glioma cells, DNA damage-associated γ-H2AX foci and the cellular distribution of Trx1 were illustrated by immunofluorescence assay. As shown in Fig. 5, γ-H2AX foci disappeared within 4 h post-IR in U-87MG cells transfected with pcDNA3.1 and scramble siRNA. In TIGAR-low-expressing cells the γ-H2AX foci remained obvious at 8 h post-IR, indicating a noticeable postpone of DDR process. Interestingly, the DDR process was notably accelerated by TrxR1 overexpression, since the fluorescent signal of γ-H2AX was vanished within 2 h post-IR. Meanwhile, IR-induced Trx1 nuclear translocation was notably enhanced by TrxR1 overexpression and diminished by TIGAR knockdown. The content of IR-induced Trx1 nuclear translocation seemed to be positively correlated with the DDR process. Most importantly, in cells treated with both TrxR1 overexpression and TIGAR abrogation, the γ-H2AX foci disappeared even later than that in control cells, which indicated a significant radiosensitization of TrxR1-overexpressing cells by TIGAR interfering.

### TIGAR inhibition radiosensitizes TrxR1-overexpressing glioma *in vivo*

**Figure 6:**
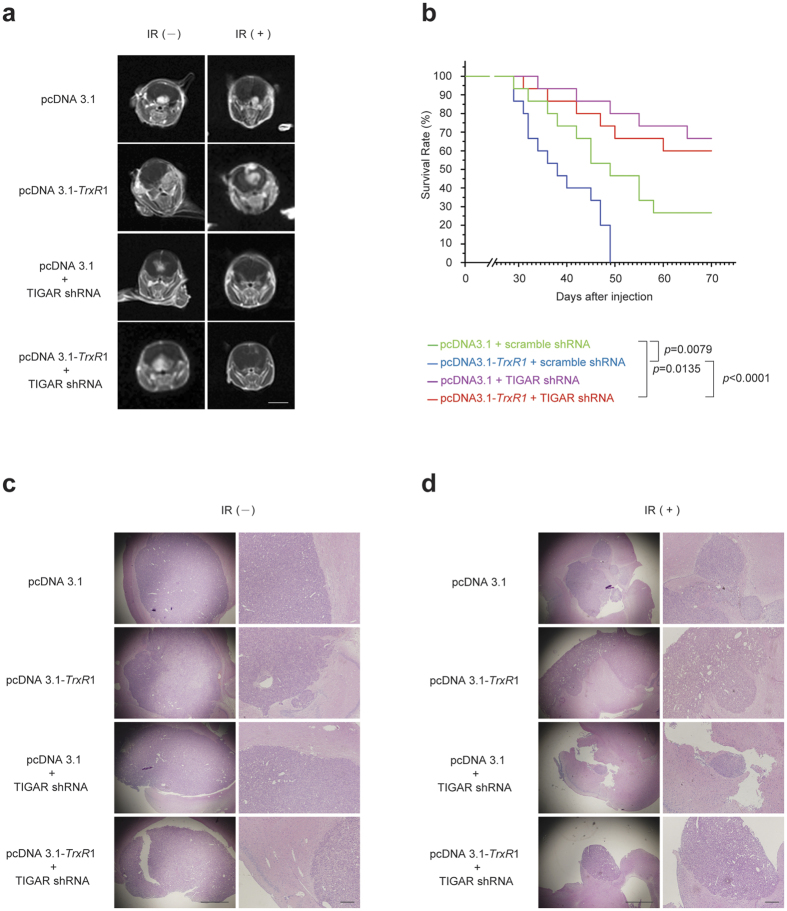
TIGAR abrogation increases the radiosensitivity of TrxR1-overexpressing glioma *in vivo*. (**a**) Axial T1-weighted images of tumour-bearing mice. At the end of brain-focalized radiotherapy, contrast enhanced MRI images of the animal brains were obtained. Left panels: sham IR, Right panels: 20-Gy fractionated IR (scale bar = 6 mm). (**b**) The Kaplan-Meier survival analysis of nude mice inoculated with U-87MG cells as indicated. Each group was composed of 15 female BALB/c nude mice. (**c**,**d**) H&E staining of xenografts with or without irradiation was performed post 20-Gy fractionated radiotherapy. Left panels: 40x magnification (scale bar = 500 μm), Right panels: 100x magnification (scale bar = 100 μm).

For *in vivo* experiments, TrxR1-overexpressing U-87MG glioma cells were transfected with interference lentivirus of TIGAR 96 h before tumour injection. Cell counting kit (CCK)-8 assay revealed that TIGAR was incapable of regulating the proliferation activity in U-87MG cells transfected with either pcDNA3.1 or pcDNA3.1-*TrxR1* (see Supplementary Fig. S6). Fifteen days after stereotactical inoculation, tumour manifestation was proven by magnetic resonance imaging (MRI) and brain-focalized irradiation was initiated. When the radiotherapy was accomplished (day 27 post inoculation), MRI was performed to investigate the size of orthotopic gliomas. As shown in Fig. 6a, 20-Gy fractionated radiotherapy resulted in more than one half of tumour reduction in the control group. However, in TrxR1-overexpressing glioma models, IR-induced tumour volume reduction was considerably diminished. Importantly, TIGAR shRNA lentivirus transfection could not only radiosensitize U-87MG xenografts but dramatically enhanced the radiosensitivity of TrxR1-overexpressing gliomas. Nearly complete tumour regression was induced by TIGAR abrogation in tumour models with or without TrxR1 overexpression. After radiotherapy, the survival curves of animals were shown in Fig. 6b. The median survival of animals harbouring TrxR1-overexpressing gliomas was 11 d shorter than that of animals in control group. Contrarily, TIGAR shRNA lentivirus transfection significantly prolonged the survival of animals suffered from TrxR1-overexpressing gliomas. Histological test indicated the morphology of orthotopic gliomas in nude mice (Fig. 6c and d). IR-induced tumour regression was notably abrogated by TrxR1 overexpression. Predictably, TIGAR shRNA lentivirus transfection significantly reduced the size of TrxR1-overexpressing glioma, indicating an effective radiosensitization (Fig. 6d).

### TIGAR low-expression inhibits Trx1 nuclear translocation in TrxR1-overexpressing glioma *in vivo*

**Figure 7:**
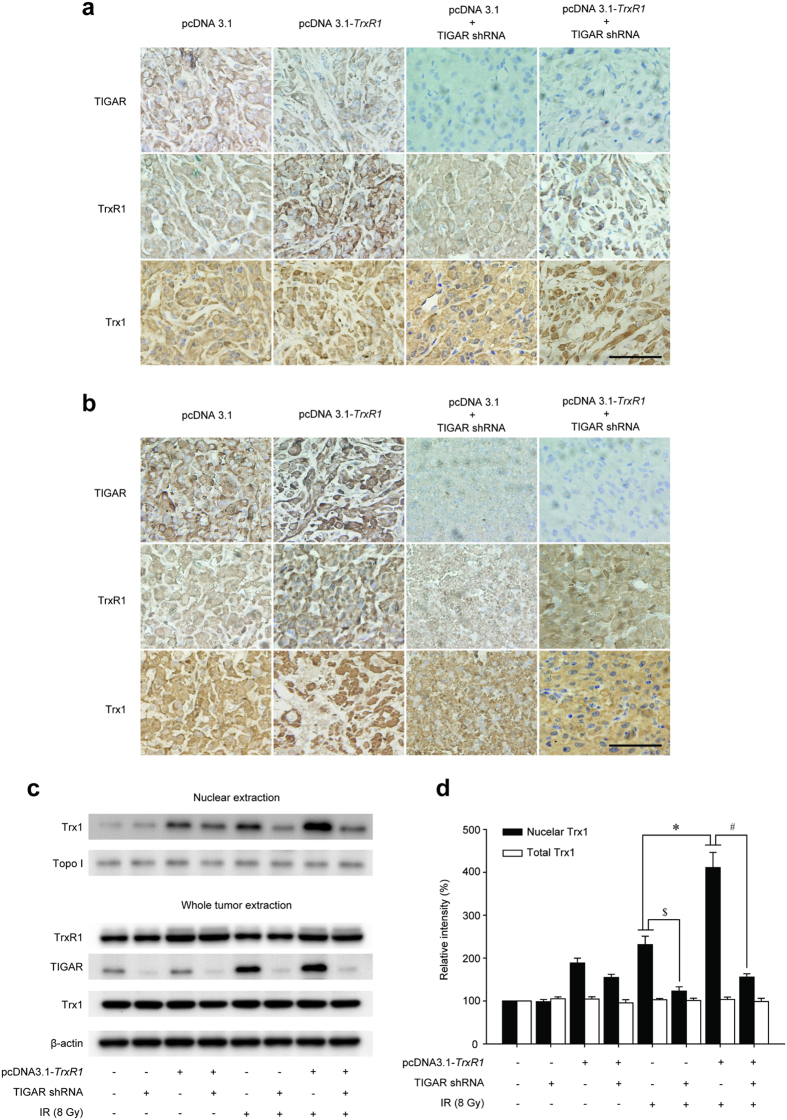
TIGAR low-expression inhibits IR-induced Trx1 nuclear translocation in TrxR1-overexpressing xenograft models. TIGAR, TrxR1 and Trx1 expression in xenografts was shown by immunohistochemistry staining (400x) performed post 20-Gy fractionated radiotherapy. (**a**) Sham IR. (**b**) 20-Gy fractionated IR (scale bar = 50 μm). (**c**,**d**) Western blot analysis of protein expression levels in freshly harvested xenografts. *p < 0.05, pcDNA3.1 vs. pcDNA3.1-*TrxR1*, ^#^*p* < 0.05, pcDNA3.1-*TrxR1* vs. pcDNA3.1-*TrxR1*+ TIGAR shRNA, ^$^*p* < 0.05, pcDNA3.1 vs. pcDNA3.1+ TIGAR shRNA.

In order to confirm the mechanism of TIGAR abrogation-induced radiosensitization of TrxR1-overexpressing glioma *in vivo*, we carried out Trx1 transport assays on freshly harvested xenografts. Before irradiation, Trx1 antibody reacted predominantly with the cytoplasm. TrxR1 overexpression alone resulted in a moderate nuclear distribution of Trx1 (Fig. 7a). After being irradiated, TrxR1-overexpressing gliomas exhibited robust staining of Trx1 throughout the nucleus, indicating a more complete nuclear transport than that in parental xenografts (Fig. 7b). Interestingly, IR-induced nuclear expression of Trx1 was significantly abolished by TIGAR knockdown regardless of TrxR1 expression, as there was little or no reaction of Trx1 antibody with nucleus in the xenografts. Moreover, TIGAR abrogated gliomas became necrosis after 20-Gy irradiation, suggesting a potentially radiosensitive effect on malignant gliomas. In order to confirm the expression levels of Trx1 Western blot assay was performed. As shown in Fig. 7c and d, IR-induced up-regulation of nuclear Trx1 was promoted by TrxR1 overexpression while suppressed by TIGAR abrogation. However, total Trx1 levels remained the same when treated with TIGAR abrogation and/or TrxR1 overexpression.

## Discussion

Malignant gliomas account for approximately 70% of new cases of malignant primary brain tumours that are diagnosed in adults in the United States each year^22^. Despite recent advances in extended-margin tumour resection, radiotherapy and targeted chemotherapies, the prognosis of malignant gliomas remains dismal^22^,^23^. Worse still, TrxR1, a widely-existing reductase is found to be up-regulated in more than one half of gliomas, which makes the prognosis even poorer^2^. As a result, numerous electrophilic compounds including some environmental toxins and pharmaceutical drugs are designed to inhibit the reduction activity of TrxR1 and lead an accumulation of oxidized Trx1^8^. However, TrxR1 inhibition alone is found to be inadequate to oxidize Trx1 in some tumour cells^24^. The reason is related to the compensatory effects of another disulfide reductase system, glutathione (GSH)-glutaredoxin (Grx) system. When TrxR1 is absent, Trx1 may get the electrons from NADPH via GSH-Grx system to maintain its reduced status and biological functions^8^,^24^. Consequently, there is a pressing need for more effective treatments targeting TrxR1-overexpressing tumour cells.

Since TIGAR is important for NADPH generation in glioblastoma cells exposed to irradiation^14^, and NADPH is essential for both TrxR1 and GSH-Grx system, it is speculated that TIGAR abrogation combined with irradiation may deplete the NADPH level in TrxR1-overexpressing glioma cells, hence abrogated the reductive activity of Trx1 without a backup role of GSH-Grx system. In this study, TrxR1-overexpressing U-87MG and T98G glioma cells were constructed to investigate the mechanisms of TrxR1-induced tumour malignant phenotype. Furthermore, TIGAR interfering was performed to radiosensitize TrxR1-overexpressing glioma both *in vitro* and *in vivo*.

Up-regulation of TrxR1 is recognized to play a critical role in cell survival and proliferation in a wide variety of tumours such as lung cancers, breast cancers and colorectal cancers^16^,^25^,^26^. Our data revealed that the proliferation rate of both U-87MG and T98G glioma cells was enhanced by TrxR1 overexpression *in vitro*. On the other hand, malignant gliomas cannot be completely eliminated surgically because of their infiltrative nature, although maximal surgical resection is undergone^22^. In this study, the invasive ability of TrxR1-overexpressing glioma cells was also examined. It was revealed that the invasive activity of TrxR1-overexpressing glioma cells was even stronger than that of control vector transduced ones. In order to demonstrate the reason why TrxR1 overexpression was closely related to the high proliferation and invasion activity in glioma cells, both the expression and nuclear translocation levels of Trx1 were investigated. Although TrxR1 overexpression played no significant role in the total expression level of Trx1, the nuclear Trx1 expression was notably increased by pcDNA3.1-*TrxR1* transfection.

Since NADPH plays an indispensable role in the Trx system, the NADPH content as well as the ROS level was detected post TrxR1 overexpressing. More than 20% increase of NADPH level was observed in TrxR1-overexpressing U-87MG glioma cells, along with a slight decrease in ROS formation. These results illustrated that the cellular redox state seemed to be more reduced by TrxR1 transfection, which might be favorable to DNA synthesis-based proliferation and the activities of invasion genes^16^,^18^,^27^,^28^.

The antioxidant capacity up-regulated by TrxR1 overexpression in glioma cells was also reflected in the enhancement of radioresistance. Both the ROS and NADPH levels post-IR revealed a relatively more reduced state induced by TrxR1 overexpression. Interestingly, IR-induced TIGAR up-regulation in TrxR1-overexpressing U-87MG cells was more obvious than that in cells being irradiated alnoe, which might be associated with a higher demand for NADPH as electron donors to maintain the reduction state of Trx1. Importantly, TrxR1-overexpressing U-87MG and T98G glioma cells were considerably re-radiosensitized by TIGAR silence. Besides, the radiosensitivity of glioma cells co-treated with TrxR1 overexpression and TIGAR knockdown was even higher than that of control cells, indicating an excellent radiosensitizing effect of TIGAR interfering.

In order to demonstrate the mechanism of TIGAR silence-induced radiosensitization in TrxR1-overexpressing glioma cells, IR-induced Trx1 nuclear translocation was examined. Western blot assays revealed that TIGAR abrogation alone was incapable of inhibiting the basal expression level of nuclear Trx1 in glioma cells with or without TrxR1 overexpression. Nevertheless, IR-induced Trx1 nuclear transport was apparently inhibited in TIGAR-low-expressing glioma cells. It was worth emphasizing that even in TrxR1-overexpressing glioma cells, IR-induced Trx1 nuclear transport was almost completely suppressed by TIGAR interfering.

It is reported that, reduced Trx1 is able to promote the transcriptional activity of p53 in nucleus, which is benefit to the nuclear redox signaling and DNA damage repair (DDR)^29^,^30^. Since TIGAR itself is transcriptionally activated by p53 in irradiated cells^14^,^31^, it could be speculated that TIGAR knockdown-induced inhibition of Trx1 nuclear translocation is able to down-regulate the transcripitional activity of p53, thereby further decreases TIGAR expression in return. Using Western blot analysis, it was determined that IR-induced TIGAR activation in U-87MG cells (wild-type p53) was completely abolished by TIGAR silence, indicating a positive feedback in TIGAR abrogation. Importantly, this feedback was also found in TrxR1-overexpressing U-87MG glioma cells, which might be associated with the excellent radiosensitizing effect.

Immunofluorescence further demonstrated the mechanism of TIGAR silence-induced radiosensitization in TrxR1-overexpressing glioma cells. Although the basal level of nuclear Trx1 was a bit higher in cells co-treated with TrxR1 overexpression and TIGAR abrogation, there seemed to be no IR-induced Trx1 nuclear transport in these cells. Moreover, the extreme level of nuclear Trx1 in cells co-treated with TrxR1 overexpression and TIGAR abrogation was significantly lower than that in their parental cells, which led to a postponed DDR process. Although TrxR1 was overexpressed, neither nuclear Trx1 expression nor radioresistance of Trx1-mutant glioma cells was enhanced at all, indicating TrxR1 regulated radiosensitivity of glioma cells was mainly dependent on Trx1 nuclear translocation.

Finally, the radiosensitizing effect of TIGAR silence *in vivo* was confirmed. In order to minimize any artifactual effects of implanting glioma cells, an orthotopic model of glioma was incorporated into this study to better mimic the clinical disease. MRI revealed that tumour proliferation was notably enhanced by TrxR1 overexpression. Meanwhile, there seem to be an enhancement of tumour invasion in TrxR1-overexpressing glioma models based on the results of hematoxylin and eosin staining. More importantly, the radiorensitivity of TrxR1-overexpressing glioma was dramatically enhanced by TIGAR interfering. The survival time of nude mice xenografted with glioma was notably shortened by TrxR1 overexpression as expected. And TIGAR abrogation significantly prolonged the survival time of nude mice regardless of TrxR1 expression in xenografts.

In conclusion, this study demonstrated the radiosensitizing effect of TIGAR silence on TrxR1-overexpressing glioma. TrxR1 overexpression predicted shorter survival time for animals suffering from xenografts. TIGAR knockdown could notably inhibit IR-induced Trx1 nuclear translocation both *in vitro* and *in vivo*, and prolonged the survival time of nude mice bearing TrxR1-overexpressing gliomas, which supported the idea that TIGAR abrogation might be a possible adjunctive therapeutic strategy against TrxR1-overexpressing glial tumours.

## Materials and Methods

### Cell culture and radiation conditions

Human glioma cell lines U-87MG and T98G were cultured in Dulbecco’s Modified Eagle Medium (Gibco, USA) with 10% fetal bovine serum (FBS) in accordance with the manufacture’s introduction. Cells were irradiated with an X-ray-radiation source at a dose rate of 1.20 Gray (Gy)/min.

### Vector construction, transfection and selection of stable cell lines

*TrxR1* was cloned using the following primers: 5′-GCAGGTACCATGTCATGTGAGGAC-3′ (sense) and 5′-GCACTCGAGTTAACCTCAGCAGCC-3′ (antisense), then subcloned into pcDNA3.1 vector, named pcDNA3.1-*TrxR1*. To establish cells stably overexpressing TrxR1, 48 hours after transfection, cells were treated with 500 μg/ml G418 (Amresco, USA) for 1 week according to the manufacture’s introduction. Glioma cells over-expressing wild-type (WT)-*Trx1* or mutant-type (MT)-*Trx1* (K81E/K82E)^15^ was established in our previous work^14^.

### TIGAR knockdown

Two small-interfering RNAs (siRNAs) matching region 115–133 in exon 6 (5′-GCAGCAGCTGCTGGTATAT-3′; TIGAR siRNA1) and region 565–583 in exon 3 (5′-TTAGCAGCCAGTGTCTTAG-3′; TIGAR siRNA2) of the human TIGAR cDNA sequence were synthesized as described previously^14^. And a scramble sequence (5′-TTACCGAGACCGTACGTAT-3′) was synthesized as a negative control. SiRNAs were transfected using Lipofectamine^TM^ 3000 (Invitrogen, Carlsbad, CA) according to the manufacture’s introduction 48 h before irradiation.

### *In vitro* invasion assay

Cell invasion assay was performed using Transwell cell culture inserts with 8 μm pores (Corning, NY, USA) in accordance with the manufacture’s introduction. The matrigel (Corning, NY, USA) was added to the inserts 2 h before cells were plated into inserts. In brief, 1 × 10^5^ cells suspended in 100 μl of serum-free medium were seeded onto the upper chamber, and 500 μl of the same medium containing 10% FBS was placed in the lower chamber. After incubation for 24 h, cells were washed with PBS, fixed with 4% formaldehyde, and stained with crystal violet for 25 min. Cells remaining on the upper surface of filter membrane were completely removed by wiping with a cotton swab before fixed. Invaded cells were then counted from six random fields under a light microscope.

### Clonogenic survival assay

Twenty-four hours after transfection, cells were plated in triplicate into six-well plates. Plates were irradiated 24 hours after plating. Fourteen days after ionizing radiation (IR), cells were fixed and stained with Giemsa. Colonies consisting of more than 50 cells were counted as a single colony.

### Subcellular fractionation and Western blot analysis

Nucleuses of glioma cells or glioma tissue samples were isolated based on the procedure described previously by Janssen and Sen^32^. Cells were pelleted by centrifugation and lysed in hypotonic lysis buffer. Following centrifugation at 16,000 × g, the pellet (nucleus) and the supernatant (cytosol) were collected respectively. Proteins were probed with antibodies specific for TrxR1 (Abcam, Cambridge, MA), TIGAR (Abcam, Cambridge, MA) and Trx1 (Epitomics, Burlingame, CA) in accordance with the manufacture’s introduction.

### Measurement of reactive oxygen species (ROS)

ROS levels were detected by cellular ROS detection assay kit (Invitrogen, Carlsbad, CA) according to the manufacture’s introduction. In brief, cells were incubating in 20, 70-dichloro-dihydrofluorescein diacetate for 30 minutes. ROS levels were measured by flow cytometric analysis (Beckman Coulter, Brea, CA).

### NADPH analysis

NADPH level was detected by NADP/NADPH quantitation kit (Biovision, Milpitas, CA) according to the manufacture’s introduction.

### Immunofluorescence

Cells were stained with primary antibodies for γ-H2AX (Abcam, Cambridge, MA) and Trx1 (Abcam, Cambridge, MA) in accordance with the manufacture’s introduction, slides were incubated for 1 h with either Alexa-488-conjugated anti-mouse IgG for visualization of foci or Alexa-555-conjugated anti-rabbit IgG for visualization of Trx1.

### Cell counting kit (CCK)-8 assays

To value cell proliferation, U-87MG cells transfected with lentiviral encoded TIGAR-specific short hairpin RNA (shRNA) and non-targeting shRNA (Hanbio Biotechnology Co., Ltd., Shanghai, China) were seeded on 96-well cell culture cluster plates (Corning, NY, USA) at a concentration of 2 × 10^3^ cells/well in volumes of 100 μl. CCK-8 reagents (Dojindo, Kumamoto, Japan) were added into each well and then incubated for 2 h at 37 °C. The absorbency was measured at a test wavelength of 450 nm according to the introduction of CCK-8.

### Xenograft model

Female BALB/c nude mice (8-week-old) were purchased from Shanghai SLAC Laboratory Animal Co., Ltd. (Shanghai, China). Animals were anesthetized using 4% chloral hydrate and underwent stereotactic injections of acutely dissociated U-87MG glioma cells into the right striatum (5 × 10^5^ cells in 5 μl of serum-free medium). The study was conducted in accordance with protocols approved by the Animal Ethics Committee of Soochow University, China.

### Radiotherapy

Animals were randomly allocated into 4 groups and were orthotopically injected with U-87MG glioma cells, TrxR1-overexpressing U-87MG cells, TIGAR shRNA lentivirus transfected U-87MG cells or U-87MG cells co-treated with TrxR1-overexpression and TIGAR shRNA lentivirus transfection, respectively. Fifteen days post tumour injection, brain-focalized irradiation (2 Gy/day, five days per week for two week) was led under anesthesia using a 6-MV X-ray linear accelerator (Siemens, Malvern, PA) at a dose rate of 198 cGy/min. Survival data was presented using Kaplan-Meier plots and analyzed using a log-rank test. The experimental protocols were approved by the Animal Ethics Committee of Soochow University, China.

### Magnetic resonance imaging (MRI)

T1 weighted MRI scans were carried out 15 days after tumour implantation to assess the tumour growth. Twenty-seven days after injection, contrast enhanced MRI (gadopentetate dimeglumine; Magnesvist, Bayer) scans were performed using a 3 Tesla (3 T) clinical MRI scanner (BrukerBiospin Corporation, Billerica, MA) with a special coil designed for small-animal imaging. The procedures for the use and care of animals were approved by the Animal Ethical Committee of Soochow University.

### Histology and immunohistochemistry

Two hours after the last time of radiotherapy (27 d post-injection), 3 mice in each group were sacrificed for histological and immunohistochemical test. In brief, anesthetized animals were perfused with PBS and 4% formaldehyde via left ventricle. The experimental protocols were approved by the Animal Ethics Committee of Soochow University, China. Brains were fixed in 4% formaldehyde for 24 h. Fixed brains were then embedded into paraffin, and 5 μm sections were prepared. These sections were stained with haematoxylin and eosin (H&E), and examined under a light microscope. Representative sections from each specimen were immunohistochemically stained with TIGAR (Abcam, Cambridge, MA, 1:500), Trx1 (Abcam, Cambridge, MA, 1:500) and TrxR1 (Abcam, Cambridge, MA, 1:500) antibodies according to the manufacture’s introduction.

### Statistical analysis

Results are expressed as means ± standard deviation in independent experiments. Differences among samples were analyzed with the one-way ANOVA by using SPSS 18.0 software. Results with a *p* value less than 0.05 were considered statistical significant.

All experimental protocols were approved by Medical College of Soochow University and all methods were carried out in accordance with relevant guidelines and regulations.

## Additional Information

**How to cite this article**: Zhang, Y. *et al*. TIGAR knockdown radiosensitizes TrxR1-overexpressing glioma *in vitro* and *in vivo* via inhibiting Trx1 nuclear transport. *Sci. Rep.* **7**, 42928; doi: 10.1038/srep42928 (2017).

**Publisher's note:** Springer Nature remains neutral with regard to jurisdictional claims in published maps and institutional affiliations.

## Supplementary Material

Supplementary Information
