# Supplementary material for: TIGAR knockdown radiosensitizes TrxR1-overexpressing glioma in vitro and in vivo via inhibiting Trx1 nuclear transport
Source: Sci Rep. 2017 Mar 24;7:42928. doi: 10.1038/srep42928 (PMC5364507; doi:10.1038/srep42928)
Supplement: Supplementary Information [file srep42928-s1.doc]

**Supplementary Information**

Title: TIGAR knockdown radiosensitizes TrxR1-overexpressing glioma in vitro and in vivo via inhibiting Trx1 nuclear transport

By: Yushuo Zhang, Fei Chen, Guomei Tai, Jiaojiao Wang, Jun Shang, Bing Zhang, Ping Wang, Baoxing Huang, Jie Du, Jiahua Yu, Haowen Zhang, Fenju Liu


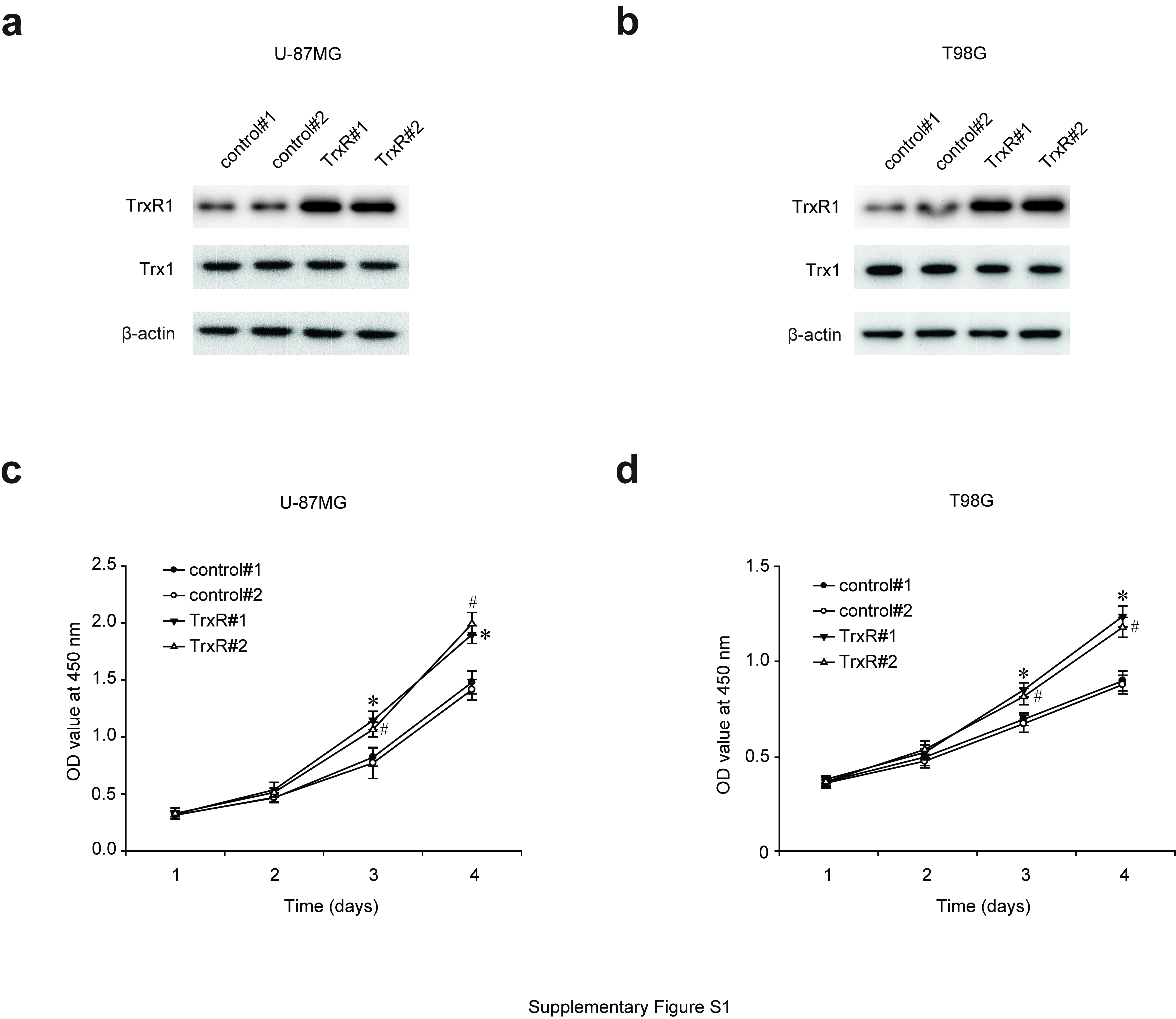





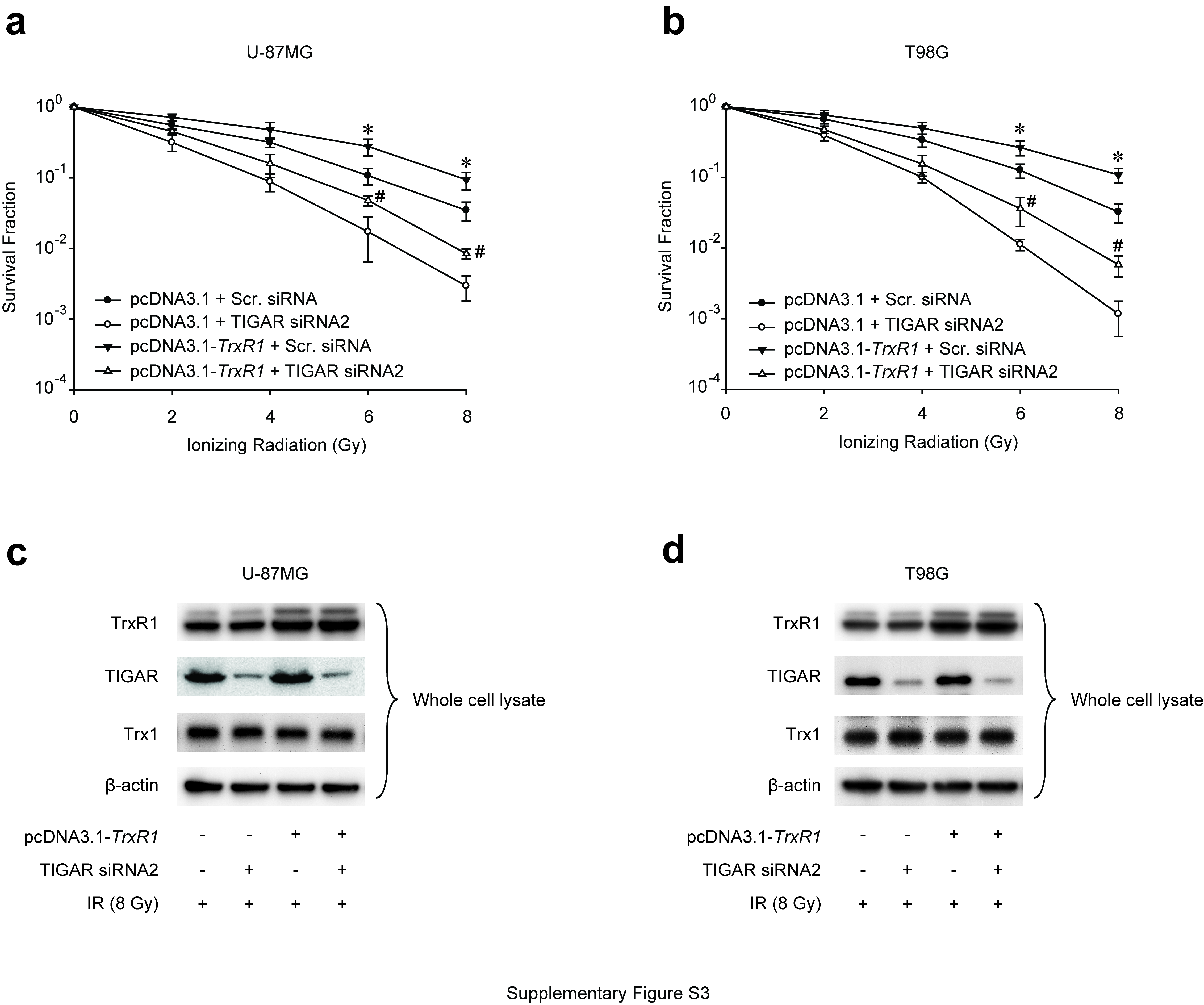


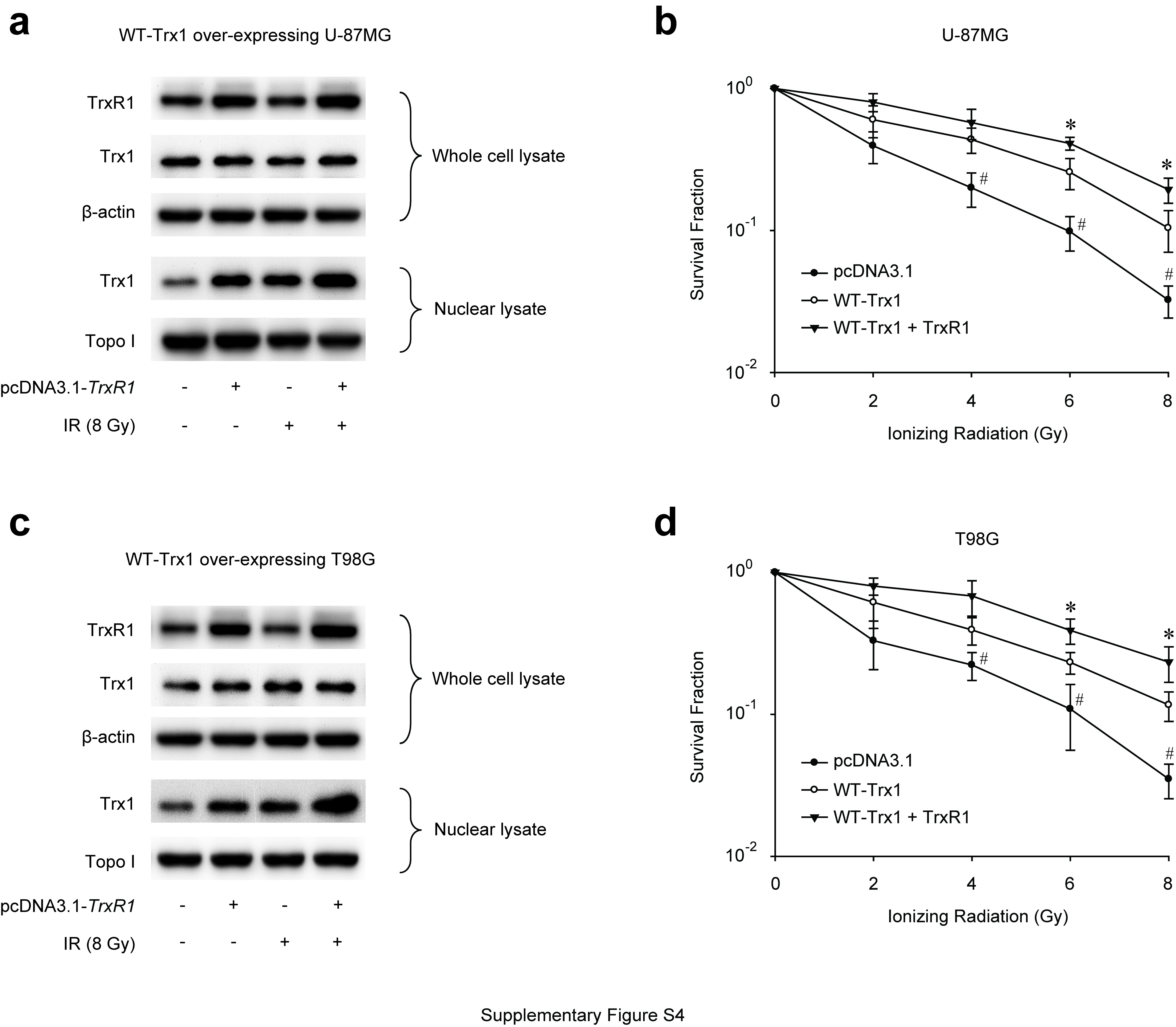


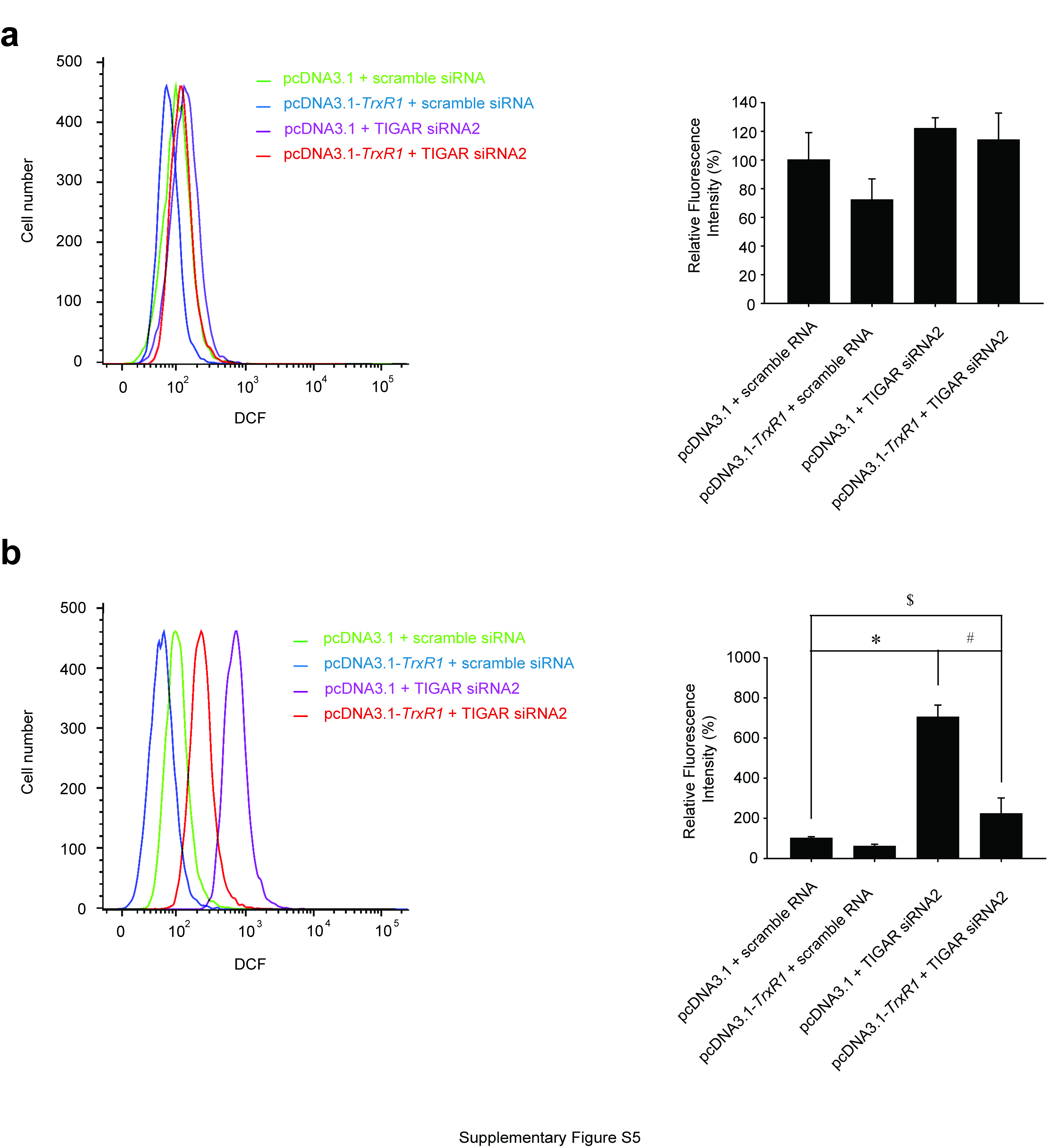


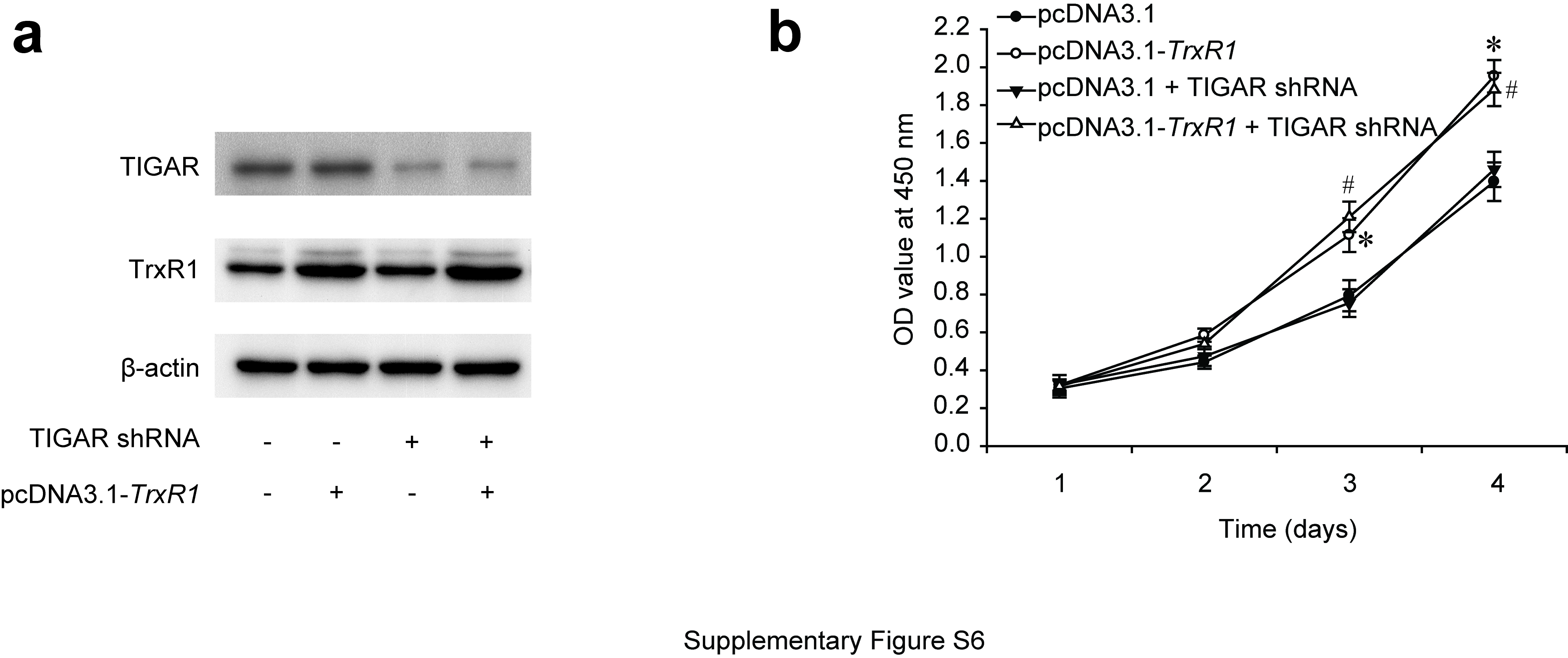


**Supplementary Figure legends:**

**Supplementary Figure 1.** TrxR1 overexpression accelerates the proliferation of glioma cells. (a) and (b) Western blot analysis of U-87MG and T98G glioma cells stably overexpressing pcDNA3.1 (control#1 and control#2) or pcDNA3.1-*TrxR1* (TrxR#1 and TrxR#2). (c) and (d) CCK-8 assays of U-87MG and T98G glioma cells stably overexpressing pcDNA3.1 (control#1 and control#2) or pcDNA3.1-*TrxR1* (TrxR#1 and TrxR#2) was performed at 24, 48, 72, and 96 h, respectively. 100 μl culture medium and 10 μl CCK-8 solutions were added to each well, followed by incubation at 37 °C for 2 h. ^*^, *p* < 0.05, control vs. TrxR#1, ^#^, *p* < 0.05, control vs. TrxR#2

**Supplementary Figure 2.** TIGAR silence is incapable of inhibiting the proliferation of glioma cells. (a) and (b) Western blot analysis of protein expression levels in U-87MG and T98G glioma cells stably overexpressing TrxR1. Cells were transfected with TIGAR siRNA1 48 h before extracted. (c) and (d) CCK-8 assays of TrxR1-overexpressing U-87MG and T98G cells treated with scramble or TIGAR siRNA1 were performed at 24, 48, 72, and 96 h, respectively. 100 μl culture medium and 10 μl CCK-8 solutions were added to each well, followed by incubation at 37 °C for 2 h. ^*^, *p* < 0.05, pcDNA3.1 + scramble siRNA vs. pcDNA3.1-*TrxR1* + scramble siRNA, ^#^, *p* < 0.05, pcDNA3.1 + TIGAR siRNA1 vs. pcDNA3.1-*TrxR1* + TIGAR siRNA1. (e) and (f) Western blot analysis of protein expression levels in U-87MG and T98G glioma cells stably overexpressing TrxR1. Cells were transfected with TIGAR siRNA2 48 h before extracted. (g) and (h) CCK-8 assays of TrxR1-overexpressing U-87MG and T98G cells treated with scramble or TIGAR siRNA2 were performed at 24, 48, 72, and 96 h, respectively. 100 μl culture medium and 10 μl CCK-8 solutions were added to each well, followed by incubation at 37 °C for 2 h. ^*^, *p* < 0.05, pcDNA3.1 + scramble siRNA vs. pcDNA3.1-*TrxR1* + scramble siRNA, ^#^, *p* < 0.05, pcDNA3.1 + TIGAR siRNA2 vs. pcDNA3.1-*TrxR1* + TIGAR siRNA2.

**Supplementary Figure 3.** TIGAR knockdown re-radiosensitizes glioma cells with TrxR1 overexpression. (a) and (b) Clonogenic capacity of TrxR1-overexpressing U-87MG and T98G cells. Scramble (Scr.) or TIGAR siRNA2 transfection was performed 48 h before irradiation. ^*^, *p* < 0.05, pcDNA3.1-*TrxR1* + Scr. siRNA vs. pcDNA3.1-*TrxR1* + TIGAR siRNA2, ^#^, *p* < 0.05, pcDNA3.1 + Scr. siRNA vs. pcDNA3.1-*TrxR1* + TIGAR siRNA2. (c) and (d) Western blot analysis of protein expression levels in U-87MG and T98G cells. Cells were transfected with TIGAR siRNA2 48 h before IR and underwent 8-Gy irradiation 2 h before being extracted.

**Supplementary Figure 4.** TrxR1 over-expression further diminishes the radiosensitivity of wild type (WT)-Trx1-overexpressing glioma cells. (a) and (c) Western blot analysis for the cell lysates of WT-Trx1 U-87MG and T98G cells. Cells were transfected with pcDNA3.1 or pcDNA3.1-*TrxR1* 48 h before IR and underwent 8-Gy irradiation 2 h before being extracted. (b) and (d) Clonogenic capacity of WT-Trx1-overexpressing U-87MG and T98G cells. Cells were transfected with pcDNA3.1 or pcDNA3.1-*TrxR1* 48 h before irradiation. ^*^, *p* < 0.05, WT-Trx1 vs. WT-Trx1 + TrxR1, ^#^, *p* < 0.05, pcDNA3.1 vs. WT-Trx1.

**Supplementary Figure 5.** TIGAR abrogation aggravates IR-induced oxidative stress in TrxR1-overexprssing glioma cells. (a) U-87M cells overexprssing TrxR1 were transfected with scramble siRNA or TIGAR siRNA2. Flow cytometric assessment of ROS production was performed 48 h post-transfection. (b) U-87MG cells were transfected with TIGAR siRNA2 48 h before IR and underwent 8-Gy irradiation 1 h before flow cytometric assessment. ^*^, *p* < 0.05, pcDNA3.1 + scramble siRNA vs. pcDNA3.1 + TIGAR siRNA2, ^#^, *p* < 0.05, pcDNA3.1 + TIGAR siRNA2 vs. pcDNA3.1-*TrxR1* + TIGAR siRNA2, ^$^, *p* < 0.05, pcDNA3.1 + scramble siRNA vs. pcDNA3.1-*TrxR1* + TIGAR siRNA2.

**Supplementary Figure 6.** TIGAR expression was not associated with proliferation in vitro. (a) Western blot assay of TIGAR and TrxR1 levels in U-87MG cells as indicated. TrxR1-overexpressing U-87MG cells were transfected with interference lentivirus of TIGAR (TIGAR shRNA) 96 h before being extracted. (b) Ninety-six hours post TIGAR silence, CCK-8 assays of U-87MG cells was performed at indicating times, respectively. ^*^, *p* < 0.05, pcDNA3.1 vs. pcDNA3.1-*TrxR1*, ^#^, *p* < 0.05, pcDNA3.1 + TIGAR shRNA vs. pcDNA3.1-*TrxR1* + TIGAR shRNA.
